# Herpes Simplex Virus Vectors for Gene Transfer to the Central Nervous System

**DOI:** 10.3390/diseases6030074

**Published:** 2018-08-14

**Authors:** Sara Artusi, Yoshitaka Miyagawa, William F. Goins, Justus B. Cohen, Joseph C. Glorioso

**Affiliations:** 1 Department of Microbiology and Molecular Genetics, University of Pittsburgh, 450 Technology Drive, Pittsburgh, PA 15219, USA; saa176@pitt.edu (S.A.); goins@pitt.edu (W.F.G.); jbc@pitt.edu (J.B.C.); 2 Department of Molecular and Medical Genetics, Nippon Medical School, Tokyo 113-8602, Japan; yoshitaka-miyagawa@nms.ac.jp

**Keywords:** neurodegenerative diseases, gene transfer, replication-defective viral vectors, herpes simplex virus type 1 (HSV-1)

## Abstract

Neurodegenerative diseases (NDs) have a profound impact on human health worldwide and their incidence is predicted to increase as the population ages. ND severely limits the quality of life and leads to early death. Aside from treatments that may reduce symptoms, NDs are almost completely without means of therapeutic intervention. The genetic and biochemical basis of many NDs is beginning to emerge although most have complex etiologies for which common themes remain poorly resolved. Largely relying on progress in vector design, gene therapy is gaining increasing support as a strategy for genetic treatment of diseases. Here we describe recent developments in the engineering of highly defective herpes simplex virus (HSV) vectors suitable for transfer and long-term expression of large and/or multiple therapeutic genes in brain neurons in the complete absence of viral gene expression. These advanced vector platforms are safe, non-inflammatory, and persist in the nerve cell nucleus for life. In the near term, it is likely that HSV can be used to treat certain NDs that have a well-defined genetic cause. As further information on disease etiology becomes available, these vectors may take on an expanded role in ND therapies, including gene editing and repair.

## 1. Overview: Neurodegenerative Diseases

Our brain is a highly complex organ responsible for cognition, learning, memory, behavior, and movement. In short, it defines who we are and informs us about our external world. The great majority of brain-related diseases and syndromes compromise particular components of brain function, with life-changing and often devastating effects on patients. Central nervous system (CNS)-related diseases have a very broad basis. Some arise early and persist throughout life as a result of defective developmental processes, but those that affect the largest segment of the population have a later onset and involve neurodegeneration. Some fifty million Americans are estimated to suffer from neurodegenerative disease [1] at a worldwide prevalence of 15% (World Health Organization, 2007: http://www.who.int/mediacentre/news/releases/2007/pr04/en/). Due to progressive aging of the global population, these numbers are expected to dramatically increase. Alzheimer’s disease (AD) alone is predicted to see a doubling in the number of affected individuals by 2030 and tripling by the end of 2050 according to the 2012 Annual Report of the Alzheimer’s Association [2]. Among others, neurodegenerative disorders include AD, Parkinson’s disease (PD), Huntington’s disease (HD), amyotrophic lateral sclerosis (ALS), frontotemporal dementia (FTD) [3,4,5] and epilepsy (Table 1). The direct cause of neurodegeneration is not always known, but these conditions are typically associated with genetic factors, such as single or multiple gene mutations or epigenetic changes, or with trauma, hormone imbalances, inflammatory processes or infectious diseases [3,6,7].

Some neurodegenerative diseases have a clear genetic basis, principally those caused by a single gene defect. These include lysosomal storage diseases (typically autosomal recessive; e.g., Gaucher disease) and trinucleotide repeat-expansion disorders (autosomal dominant) such as Huntington’s disease and several spinocerebellar ataxias (SCAs) (Table 1). Others have complex, but recognizable genetic defects. For example, early-onset AD has been linked to mutations in the genes encoding amyloid precursor protein (APP) and the presenilins (PS-1 and PS-2) involved in amyloid-β processing [3,4], while the ε4 allele of the apolipoprotein E gene has been identified as a risk factor for late-onset AD [8,9,10]. Likewise, some 10% of PD cases are strongly associated with α-synuclein (SNCA) and microtubule-associated protein tau (MAPT) gene alterations [3,11,12], and approximately 3% of cases are associated with missense mutations in the leucine-rich repeat kinase 2 (LRRK2) gene [13]. Very recently, somatic mutations in the ATP6V1A gene have been identified as a major factor in the development of encephalopathy with epilepsy [14]. However, our knowledge of the contributing factors and affected pathways in complex neurodegenerative disorders remains deficient. 

While specific brain regions tend to be prominently affected in brain neurodegeneration, multiple cell types distributed throughout the brain are often impacted as well and brain degeneration is therefore generally of a global nature, making the design of treatment strategies challenging. In addition, even diseases with known genetic causes are often accompanied by inflammatory responses that contribute to the degenerative process, and changes in metabolic processes carried out by mitochondria, for example, can directly affect neuronal cell function or viability [15,16,17]. Current approaches are mainly focused on alleviating symptoms using small molecules, such as L-DOPA to treat early-onset Parkinsonism, but fail in the long-term and do not address the fundamental disease causes. Often, these treatments suffer from poor drug bioavailability due to the blood brain barrier (BBB) and may impact cells not directly involved in the disease process [18]. Peptides as delivery systems to the CNS constitute an interesting alternative. While peptides face similar bioavailability issues as traditional small molecules and can readily undergo metabolic degradation and clearance in the liver, they can be designed for specific receptor-mediated interaction with and transport through the BBB, thus enabling efficient cargo delivery to the brain [19,20,21]. A wide variety of peptide modifications continue to be developed and tested for improved performance in the delivery of therapeutic agents to the CNS, including strategies to enhance general stability and resistance to proteolytic enzymes (e.g., cyclization, dimerization, amino acid substitutions), and to optimize lipophilicity, specificity, and target-cell penetration [19,20,21,22]. Overall, although peptide carriers show significant promise for the treatment of a multitude of diseases [23], they do not provide cures. Given the slow progression of many NDs, it is clear that neither such pharmacological, nor electrical interventions will be satisfactory for these conditions in the long term. As the genetic basis of brain disease comes into sharper focus, the potential of genetic intervention is therefore becoming increasingly attractive. 

## 2. Background: Gene Therapy Strategies, Viral Vectors and Applications

Gene therapy is defined as a therapeutic intervention through the delivery of an exogenous gene or genes. In 1990, the first human gene therapy trial, aimed at correcting adenosine deaminase deficiency (ADA) in T-lymphocytes of a four-year-old girl using a retroviral vector [27,28,29], showed a positive response although continuous protein therapy was still required. Despite several significant hurdles and setbacks since, including the death of a patient in 1999 following injection of a recombinant adenoviral vector to treat the metabolic disease ornithine transcarbamoylase deficiency (OTC) [30,31], gene therapy has come into its own as a promising therapeutic tool that may become the standard of care for certain diseases. While at first gene therapy was almost exclusively focused on rare monogenic disorders, due to an expanding repertoire of gene delivery vehicles and technologies, it is now increasingly under consideration for a broader array of diseases [29,30]. The approval of the non-replicative adenoviral vector Gendicine for the treatment of head-and-neck cancer in 2003 [32], the adeno-associated virus (AAV)-derived vector Glybera targeting a lipoprotein lipase deficiency in 2012 [33], and AAV vector-based Luxturna for retinal dystrophy (Lebers congential amaurosis) in 2017 [34] confirmed the potential of this field [30]. Based on promising results from phase I/II trials, two AAV-based Factor IX gene delivery vectors for the treatment of hemophilia B (SPK-9001, OMT-061) have been designated breakthrough therapies by the US FDA [35] (http://ir.sparktx.com/news-releases/news-release-details/spark-therapeutics-and-pfizer-present-updated-preliminary-data). Furthermore, a heavy emphasis on cancer in the gene therapy field has resulted in FDA approval of genetically altered T cells (CAR-T) as an effective therapy for lymphoblastic leukemia (Kymriah, Novartis) in 2017 (https://novartis.gcs-web.com/novartis-receives-fda-approval-for-KymriahTM) and an engineered (‘oncolytic’) herpes simplex virus, T-Vec (Imlygic, Amgen), in 2015 (http://www.multivu.com/players/English/7414056-amgen-imlygic-fda-approval/) for the treatment of inoperable melanoma. Most recently, breakthrough advances in gene-editing technologies bring the prospect of precise human genome editing closer to reality, allowing not only ectopic gene introduction at a favorable chromosomal site, but also specific inactivation of defective genes and correction of single or multiple deleterious gene mutations [36,37]. 

For decades, the gene therapy field has relied on the development of suitable delivery vehicles and a good portion of this developmental work has centered on the creation of viral vectors. The bar for viral vectors is high since they must be safe and nonpathogenic, amenable to consistent manufacture, and capable of delivering a therapeutic payload to the correct tissue and cell type. The therapeutic gene must be expressed not only at the appropriate level, and thus be regulated either by transcriptional or post-transcriptional mechanisms, but also for an appropriate length of time in order to achieve maximal benefit [38]. In addition, the therapy must persist without damage to the transduced cells or recruitment of immune responses that could eliminate corrected cells and shut down transgene expression. The most common systems used for gene therapy are adenovirus, gamma-retrovirus, lentivirus, adeno-associated virus and herpes simplex virus-based vectors, ranging in genome size from ~5–150 kb. 

Originally developed for clinical use in the CNS [38], AAV appears to be our most promising gene delivery system thus far since it is not a human pathogen, requires co-infection with a helper virus in order to replicate, and can persist in an extrachromosomal state, particularly in nondividing cells [39,40]. With a genome size of ~4.7 kb, AAV is among the smallest viruses used for gene therapy and has proven particularly adept at delivery of small gene cassettes (<5 kb) to wide areas of many tissues, including brain [41]. However, systemic use of AAV may prove harmful due to immunotoxicity and hepatic genotoxicity at high doses, and thus targeted delivery is likely to be critical for safety [42,43,44,45].

Retroviruses have proven to be effective in ex vivo therapies involving bone marrow stem cell transplants [46], while adenoviruses and herpes viruses have been largely used in cancer therapies, taking advantage of the ability of these viruses to both lyse tumor cells and induce antitumor immunity, especially when armed with immune-modulatory genes [47,48]. The major concern for retroviruses and lentiviruses relates to their integration into the host genome. Integration could be seen as an advantage to accomplish a long-term outcome, but it also involves significant risk of insertional mutagenesis promoting cancer development [49,50,51]. Adenoviruses are characterized by a lack of integration capability as part of their life cycle, but are among the viral vector categories showing high toxicity levels and induction of a robust host immune response [52,53], thus requiring further studies and improvements. Derivatives of many other viruses, including a number of single-stranded RNA viruses [54], are under development as oncolytic vectors [55]. Early attempts to treat complex brain diseases, such as PD, with AAV vectors expressing genes in support of dopamine production in the substantia nigra, have met with limited success [56,57]. Indeed, treatment of symptoms alone most likely will not prove adequate and disease correction may well require multiple genes delivered to individual cells. Our view is that high capacity vectors that can accommodate large and multigene cassettes, such as HSV-1 derived viruses, will be essential to the treatment of complex genetic diseases, particularly in the brain. 

## 3. HSV-1- and HSV-1-Based Viral Vectors

Over the last 30 years, considerable progress has been made in the development of gene transfer vectors based on HSV. HSV is a neurotropic, double-stranded DNA virus that was found many decades ago to persist in the peripheral nervous system [58,59,60,61]. Since then, many studies of HSV latency have been carried out to determine the state of the genome, gene expression and the role of immunity in maintaining the latent state. Several features make this herpes virus attractive as a viral vector and particularly suitable for the treatment of nervous system diseases [38,62]. First, HSV shows tropism for a wide variety of cell types with high infectivity for both dividing and nondividing cells. Second, it expresses over 80 different genes, many of which are not essential for its replication cycle. HSV therefore has the potential to carry a substantial payload, allowing the insertion of multiple or very large transgenes in highly defective vectors described below. Third, the latent HSV genome does not integrate into cellular DNA, but remains episomal as a closed circular molecule [63,64,65], thus avoiding the risks of insertional mutagenesis. HSV has the interesting feature of intra-axonal transport that allows the virus to infect peripheral nerve terminals of the epithelium, followed by rapid retrograde transport to the nerve cell body without exposure to the immune system. Delivery of the viral DNA to the nucleus results in latency in which the virus lytic genes are silenced. HSV reactivation from latency is common and generally follows nerve damage or compromise of the immune system [58]. While the details of the molecular events that either induce latency or allow virus reactivation are not completely understood, it is clear that HSV can persist in neurons for life. During latency, the viral genome is transcriptionally silent with the exception of the latency-associated transcript (LAT) gene responsible for the production of a stable, noncoding intron processed from a long, unstable primary transcript that also contains several microRNA sequences [66,67,68]. In non-neuronal cells that come into contact with HSV, infection results in lytic replication and cell death. The main challenge in the development of HSV vectors for CNS disorders relates to the establishment of a latent-like infection that allows durable therapeutic gene expression without activation of viral genes that code for cytopathic proteins or are required for reactivation. Two main categories of HSV-based vectors have been generated to achieve these goals: HSV-1 amplicon vectors and replication-defective HSV-1 vectors.

## 4. Amplicons as Viral Vectors: Pros & Cons

**Figure 1:**
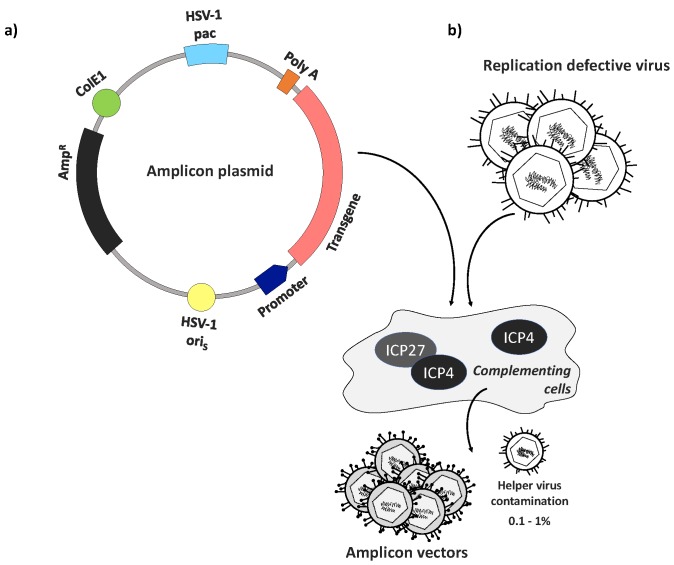
Amplicon plasmid structure and vector generation. (**a**) Amplicon plasmids typically combine the presence of a transgene cassette with the ampicillin resistance gene (AmpR, black), a bacterial origin of replication (ColE1, green), a single HSV origin of replication (oriS or oriL, yellow), and an HSV-1 packaging (pac) signal (light blue). The transgene (pink) is accompanied by a promoter (dark blue) and polyA signal (orange) for expression in mammalian cells. The use of minicircle plasmids lacking bacterial sequences has been shown to increase amplicon transgene expression levels and duration [74]; (**b**) Basic procedure of amplicon vector production using a replication-defective helper virus and cells that complement essential viral gene deficiencies. Low-level cross-contamination of the final product with helper virus remains a concern [72].

HSV amplicon vectors are minimal HSV vectors that rely on a full complement of helper virus functions for their production, but stand out for their unparalleled payload capacity (~150 kb). Like other HSV vectors, amplicon vectors remain extrachromosomal and therefore pose no risk of insertional mutagenesis [69,70]. They are essentially regular HSV virions that possess the same properties as typical HSV-1 particles, including structure, tropism and immunogenicity, but package a genome-length amplicon plasmid concatemer instead of a functional HSV genome. Standard amplicon plasmids consist of an E.coli origin of replication (ColE1), an antibiotic resistance (AmpR) gene, a transgene expression cassette of up to 150 kb in size, a single HSV-1 origin of replication (oriS or oriL), and an HSV-1 cleavage/packaging signal (pac) [70,71,72] (Figure 1a). Due to this simple design lacking any viral protein-coding genes, the amplicon life cycle and production are completely dependent on the presence of a helper virus, typically a replication-defective HSV-1 to minimize amplicon stock contamination with replication-competent HSV; complementing cells are used to enable helper virus replication and thereby amplicon replication and packaging (Figure 1b). The amplicon DNA replication process occurs via a theta-mechanism giving rise to a series of amplicon-plasmid tandem repeats the length of the HSV genome [69], thus providing an extraordinary capacity to accommodate multiple genes of average-size or very large transgenes. The absence of viral protein expression makes these vectors completely nontoxic for target cells. However, the total reliance on helper viruses for amplicon production and packaging make this platform challenging to manufacture and validate. The production method ultimately leads to cross-contamination of amplicon stocks with variable amounts of helper virus particles that are potentially cytotoxic and immunogenic [69]. Many attempts have been made to achieve high-titer and entirely helper virus-free stocks, but even consistently low cross-contamination (0.1–1%) was observed to cause cytotoxicity [69,72,73]. Despite the remarkable potential of HSV amplicon vectors for a broad spectrum of applications, including both gene therapy and vaccine development, persistent concerns about their safety and the difficulties involved in their cGMP manufacturing have encouraged the continuing development of an alternative category of HSV vectors, as described below.

## 5. Replication-Defective HSV Vectors

**Figure 2:**
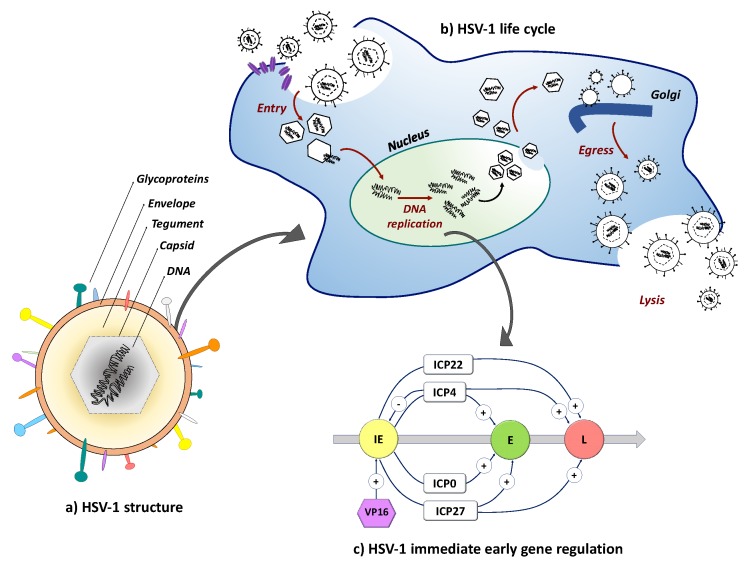
Schematic representation of the HSV-1 virion structure and lytic life cycle. (**a**) HSV-1 is composed of an envelope containing multiple glycoproteins that function in receptor-dependent viral entry and spread, a tegument, and a capsid containing the 152-kb linear double-stranded DNA genome; (**b**) HSV-1 entry by envelope fusion with plasma or endosomal membranes is initiated by viral glycoprotein D (gD) interaction with a cognate receptor (purple) and executed by glycoprotein B (gB) via signal transduction through a complex of gH and gL [78,79]. Released capsids inject their viral DNA into the nucleus where replication and encapsidation take place. Complex envelopment, de-envelopment and re-envelopment steps at nuclear and Golgi membranes ultimately result in cell lysis and virion release; (**c**) Cascade regulation of HSV gene expression. The three kinetic categories of HSV-1 genes, the immediate early (IE), early (E) and late (L) genes, are illustrated along with their expression dependence on prior viral gene products.

While HSV establishes a life-long quiescent (latent) infection in neurons, reactivation from latency can occur with potentially severe consequences. Reactivation requires replication competence and thus it was quickly recognized that disabling virus replication genes would prevent reactivation. HSV genes are expressed in an interdependent sequence referred to cascade regulation that distinguishes three separate gene categories [75,76,77]. The immediate early (IE) genes are expressed first under control of a specific enhancer recognized by VP16, a virion component delivered to the nucleus during HSV infection (Figure 2). The IE genes encode infected cell protein (ICP)0, ICP4, ICP22, ICP27 and ICP47. With the exception of the ICP47 gene, the IE genes control the expression of the early (E) and late genes (L) that encode the necessary functions for viral genome replication and the virion structural proteins, respectively. Capsid assembly occurs in the nucleus, along with acquisition of the viral tegument proteins surrounding the capsid. Subsequent acquisition of the virus envelope is accomplished through several membrane exchanges involving budding from the nuclear membrane and trafficking through the Golgi apparatus. Virus release involves cell lysis (Figure 2).

Among the five HSV-1 IE genes, two are essential to complete the virus life cycle. ICP4 displays major regulatory functions throughout virus replication [80,81] while ICP27 is essential for the processing of viral mRNAs [82,83]. Given the crucial role of these genes in the induction of the HSV-1 lytic replication cycle, HSV vectors were first deleted for one or both to render the virus replication defective. Production of vectors deleted for ICP4 and ICP27 requires complementation using engineered cell lines that express the deleted genes in trans from their virion-inducible cognate promoters [81,84,85]. Although ICP0 and ICP22 are non-essential, their products regulate cellular innate resistance pathways and thereby enable efficient virus growth [86,87]. Vectors deleted for ICP4 alone, while replication defective, were found to be highly cytotoxic [81,88], prompting the removal of additional IE genes [87,89]. Among the various double-deletion mutants tested, the aforementioned ICP4/ICP27 double deletion mutant virus provided the most favorable toxicity profile [84,90]. While additional deletion of the ICP22 gene further reduced cytotoxicity, these vectors displayed growth defects on ICP4/ICP27-complementing cells. Instead, removal of the VP16-responsive TAATGARAT motif from the ICP22 promoter/enhancer proved to be preferred over a complete gene deletion as it changed the gene’s expression kinetics to that of an early gene, remaining silent in non-complementing cells but active in the post-IE stages of vector replication in ICP4/ICP27-complementing cells and thereby enhancing vector production [86]. ICP47 mutations did not impact virus replication in vitro since this protein functions to prevent transporter associated with antigen processing (TAP) loading of antigenic peptides onto class I MHC molecules and thus interferes with immune recognition of virus-infected cells [91]. Deletion of ICP0 eliminated abundant remaining cytotoxicity of multi-IE gene deletion mutants but also profoundly impaired virus production [86,90,92]. Consistent with the results of a transfection study of individual IE genes [93], these results indicated that ICP4, ICP22, ICP27 and ICP0 are cytotoxic proteins that, in the absence of disabling gene mutations, will be expressed from replication-defective vectors, causing cell death. Accordingly, inactivation of all of these genes was predicted to provide a non-toxic vector backbone. 

Due to the extreme toxicity of ICP0 [86], the establishment of complementing cells to produce recombinant viruses defective for expression of ICP0 and the essential IE genes was a major challenge. Using very low levels of ICP0 expression in cis or in trans [94,95], these viruses could eventually be grown to practical titers, providing the opportunity to test their characteristics and potential as gene transfer vectors. Recombinant *d*109 was constructed to eliminate all IE gene expression and was found to establish a latent-like state on infection of non-neuronal cells without detectable adverse effects on cell viability [92]. However, expression of an inserted reporter gene from a foreign promoter was dramatically reduced compared to less debilitated viruses. The demonstration that transgene expression could be rescued by subsequent expression of ICP0 in trans underscored the key dilemma in HSV vector development that has confounded efforts to advance HSV as a widely applicable gene transfer platform: ICP0 is essential for vector payload expression but kills most cell types. As an exception, ICP0 is better tolerated by sensory neurons although overexpression is toxic [96,97].

During latency, the extrachromosomal HSV genome is transcriptionally silent with the notable exception of the latency-associated transcript (LAT) gene. An unusual enhancer-like region overlapping the 5′ end of the LAT primary transcript (LAP2 or LAT P2) [98,99] was shown to enable long-term transgene expression in peripheral neurons from a foreign promoter that is normally silenced within days in a replication-deficient HSV background [99,100,101], leading to attempts to express transgenes from the LAT regulatory region in the absence of damaging IE gene activity. An interesting example was reported in 2001 by Lilley and coworkers [102]. These authors combined deletions of the ICP4 and ICP27 genes with an inactivating mutation in the VP16 gene and reported undetectable levels of the 5 IE proteins in non-complementing cells infected at high multiplicity. Virus production was made possible by the introduction of a third complementing gene, encoding the VP16 homolog of equine herpesvirus, into ICP4/ICP27-complementing cells. Insertion into the virus genome of the LAT P2 region flanked on both sides by divergent reporter expression cassettes relying on different foreign promoters provided reporter gene expression for weeks in cultured Vero cells, rat dorsal root ganglion (rDRG) cells, and in CNS neurons in vivo. In contrast, an ICP4+ version of the virus caused pronounced toxicity in rDRG cultures and transgene expression in the CNS was detected only through the first week post-infection. However, the production of low levels of ICP0 and ICP22 could not be excluded, raising the potential for long-term toxicity. Moreover, the possibility of compensatory mutations restoring VP16 activity or loss of the VP16-inactivating short palindromic insertion in the VP16 gene during virus stock preparation was not addressed.

**Figure 3:**
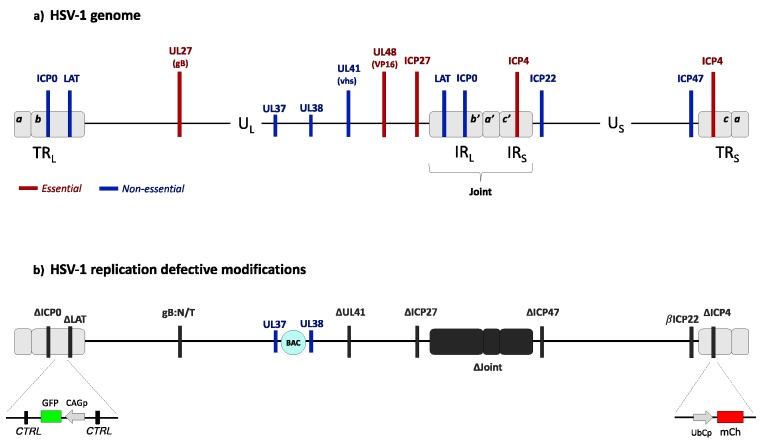
Genome structures of wild-type HSV-1 and a replication defective derivative. (**a**) Wild-type HSV-1 genome organization. The HSV-1 genome is a 152-kb-long double-stranded DNA comprising a unique long (U_L_) and a unique short (U_S_) region separated by a joint region composed of two internal regions (IR_L_ and IR_S_) repeated in inverted orientation at the genome termini (TR_L_ and TR_S_). The genome codes for at least 80 essential and nonessential genes. The locations of genes referred to below are indicated in red (essential) or blue (nonessential). (**b**) Diagram of an advanced HSV-1-based gene transfer vector [90]. The vector genome is deleted for the immediate-early genes ICP4, ICP27 and ICP0, the promoter region of the ICP47 gene, the VP16 binding site in the promoter of the ICP22 gene causing regulation as an early gene (βICP22), a large portion of the LAT gene, and the complete joint region. Additional deletion of the UL41 gene has been reported to enhance transgene expression in a cell type-dependent manner [104]. UL37 and UL38 embed the bacterial artificial chromosome (BAC) genes. Inserted reporter gene cassettes are illustrated as follows: the mCherry (mCh) gene under the control of a ubiquitin C promoter (UbCp) at the site of the ICP4 gene deletion in TR_S_, and a GFP gene controlled by a modified actin promoter (CAGp) in the LAT intron. These vectors also contain a mutant glycoprotein B gene (gB:N/T) shown to promote efficient viral entry [113]. Two regions of repeated CTCF-binding motifs surrounding the intact LAT promoter/enhancer are indicated (CTRL).

To circumvent these potential safety concerns, we and others have explored strategies to promote transgene expression from IE gene-inactive vectors deleted for the ICP4, ICP27 and ICP0 genes and minimally the VP16 response elements of ICP22 and ICP47 (Figure 3). Based on the observation that U2OS cells can complement certain functions of ICP0 [103], we were able to grow these viruses on ICP4/ICP27-transduced U2OS cells [90]. Our studies demonstrated prolonged, non-toxic transgene expression from a foreign promoter inserted directly downstream from the LAT promoter/enhancer region in non-neuronal cells. Interestingly, although expression from the LAT region was transient in rDRG cultures and largely undetectable in mouse hippocampus [104,105], reporter expression from the deleted ICP4 locus without adjacent LAT regulatory elements persisted for months in mouse hippocampus [106]. Furthermore, we observed that this expression could be further enhanced by deletion of the virion host shutoff gene (vhs or UL41) [104]. Studies by Harkness et al. [107], using their IE-inactive *d*109 vector, showed that genes near or within the long viral inverted repeat regions remained more active than genes in the unique regions of the largely quiescent genome in cultured trigeminal ganglion neurons. Several laboratories have studied the functions of a series of CTCF-binding regions in the HSV genome and noted the apparent association of these sites with the five IE genes and the LAT gene that are also located within or near the long viral repeats [108,109,110,111,112]. CTCF (CCCTC-binding factor) has a range of transcription regulatory activities and several of the CTCF-binding elements of HSV can act as insulators, boundaries between active and inactive chromatin. Thus, these elements are believed to play a key role in regulating IE gene expression and separating the latency-active LAT region from the remainder of the viral genome during latency. CTCF binding sites can also pair to establish long-range DNA loops, thereby potentially coordinating the expression of distant genes without effect on the intervening genes. Our vectors are deleted for multiple CTCF binding sites, but retain two sites straddling the deleted ICP4 locus as well as two sites associated with the LAT gene. It is conceivable, therefore, that the substantially altered landscape of CTCF binding sites in our vectors promotes transcriptional activity in the ICP4 locus while silencing the LAT locus in CNS neurons. Interestingly, these same modifications may be responsible for the opposite outcome observed in non-neuronal cells [90]. Current studies in our lab are aimed at gaining a better understanding of the roles and interactions of the remaining CTCF elements in our vectors and at defining the potential of non-HSV insulators to selectively promote transgene expression, particularly in the CNS [105].

## 6. Conclusions

Neurodegenerative diseases pose a significant threat to quality of life and are of growing concern as the population ages. NDs add a major cost burden to the healthcare system, particularly since disease onset is slow but severity increases over time, eventually leading to death. Although the genetic basis for many of these diseases is yet to be fully defined, genetic intervention now appears possible and applicable gene delivery tools are under development. Among these, we have created HSV vectors with high payload capacity and an ability to provide durable transgene expression in brain neurons without expression of viral genes. These vectors are deficient for expression of the IE genes, preventing the induction of inflammation, neuronal damage or perturbation of nerve cell function. Considerable effort has gone into devising methods for vector production and validation of vector integrity. Using related ICP0+ vectors, we have developed promoter systems for regulated transgene expression in sensory nerves for the treatment of chronic pain models [114], but we do not yet know whether promoter regulation will be preserved in CNS neurons in the absence of ICP0. Over the years, significant efforts have been devoted to the retargeting of HSV vectors to achieve selective transduction of specific cell types, primarily cancer cells through growth factor receptors [115,116,117,118,119]. These technologies will be applied to our nontoxic CNS vectors. Finally, we emphasize that combinations of gene therapy and gene editing represent the future of the field, including the use of large vectors such as HSV to carry out homologous repair of defective genes, an important concept for dominant genetic problems. While we are in the early days of devising gene therapy methods for the brain, such strategies are inevitable and will undoubtedly provide treatment possibilities in the future.

## Figures and Tables

**Table 1:** Prominent neurodegenerative diseases [4,22,24,25,26].

Disease	Prevalence (USA) *^a^*	Gene Mutations *^b^*	Mechanism/Symptoms	Current Treatment
Alzheimer’s disease (AD) https://www.alz.org	5.3 × 10^6^	APP APOE PSEN1 and PSEN2	Deposition of ß-Amyloid (Aß) plaques and microfibrillary tangles (Tau), neuronal cell death, memory loss	- Cholinesterase inhibitors (Aricept, Exelon) - NMDA-R antagonist (Namzaric, Memantine) - Dementia care facilities
Parkinson’s disease (PD) http://parkinson.org/	1 × 10^6^	SNCA MAPT PRKN DJ1 PINK1 LRRK2	Synucleinopathy with neuronal cell death, decreased dopamine synthesis, bradykinesia and dyskinesia	- Levodopa MAO-B inhibitors - COMT inhibitors - Dementia care facilities - Deep brain stimulation
Huntington’s disease (HD) http://hdsa.orghttps://chdifoundation.org	3 × 10^5^	HTT	Cognitive and psychiatric disorders, impaired movement	- Mood stabilizers - Physical, speech and psychological therapy
Amyotrophic lateral sclerosis (ALS) http://www.alsa.org https://www.ninds.nih.gov	1–3 × 10^5^	SOD1 ALS2 FUS TARDBP	Impaired RNA processing, gene regulation and expression, protein folding and homeostasis. Motor neuron degeneration	- Riluzole (Rilutek) - Edaravone (Radicava)
Frontotemporal demetia (FTD) https://www.theaftd.org	2–3 × 10^5^	MAPT C9ORF72 VPC CHMP2B GRN	Impaired tau production and function, change in behavior, movement and language dysfunctions, memory loss	- Symptoms relief - Antidepressants, mood stabilizers
Spinocerebellar ataxia (SCA) https://ghr.nlm.nih.gov https://www.orpha.net https://ataxia.org	1.5 × 10^5^	SCA family ATXN1	Progressive movement, speech and cognitive problems	- Symptoms relief - Physical therapy

*^a^* Estimated cases per total USA population of ~326 × 10^6^. http://www.ohsu.edu/xd/health/services/brain/in-community/brain-awareness/brain-health/disease-statistics.cfm, https://www.cdc.gov/mmwr/pdf/ss/ss6307.pdf; *^b^* Causative or associated.
